# Role of HHV-6B Infection in Mesial Temporal Lobe Epilepsy

**DOI:** 10.15844/pedneurbriefs-29-5-7

**Published:** 2015-05

**Authors:** John J. Millichap, J. Gordon Millichap

**Affiliations:** 1 Division of Neurology, Ann & Robert H. Lurie Children's Hospital of Chicago, Chicago, IL; 2 Departments of Pediatrics and Neurology, Northwestern University Feinberg School of Medicine, Chicago, IL

**Keywords:** Human Herpesvirus 6, Temporal Lobe, Mesial Temporal Sclerosis

## Abstract

Investigators from Fujita Health University, Toyoake, and National Epilepsy Center, Shizuoka, Japan, studied the pathogenic role of HHV-6B in patients with mesial temporal lobe epilepsy (MTLE).

Investigators from Fujita Health University, Toyoake, and National Epilepsy Center, Shizuoka, Japan, studied the pathogenic role of HHV-6B in patients with mesial temporal lobe epilepsy (MTLE). Of 75 intractable MTLE patients, 52 had mesial temporal sclerosis (MTS) and 23 were non-MTS patients. Resected samples of hippocampus, amygdala, and mixed samples of amygdala and uncus were examined by real-time polymerase chain reaction (PCR) and reverse-transcriptase PCR to detect viral DNA and messenger RNA (mRNA), respectively. Detection of HHV-6 DNA was higher in MTS patients than non-MTS patients. Of 9 herpes viruses analyzed, HHV-6 was the most frequently detected. DNA was determined in 12/27 HHV-6 DNA-positive samples and no HHV-6B mRNA were detected in all samples. In MTS patients, expression of monocyte chemotactic protein-1 and glial fibrillary acidic protein were significantly higher in the amygdala samples with HHV-6 DNA than those without viral DNA. The number of prolonged febrile seizures early in life was higher in the MTS patients than the non-MTS patients. HHV-6B may play an important role in the pathogenesis of MTS via modification of host gene expression. Latent infection rather than reactivation of HHV-6 probably contributes to the development of MTS. [1]

COMMENTARY. Prolonged febrile seizures or febrile status epilepticus (FSE) are associated with an increased risk of MTS and TLE, the subject of an ongoing, prospective multicenter study, the FEBSTAT study [2]. In 1964 and 1968, Falconer MA, Neurosurgeon at the Maudsley Hospital, London, UK, investigating the etiology of TLE, reported 13 (28%) of 47 cases with a history of infantile convulsions ascribed to fever [3, 4]. In comparison, 7 (15%) had a history of difficult birth. As early as 1956, Cavanagh and Meyer noted the high incidence of febrile convulsions preceding onset of TLE [5]. In the recent FEBSTAT study, HHV-6B viremia is reported in 54 of 169 subjects (32%) at the time of FSE [2].

A relationship between MTS and a history of febrile seizures and HHV-6B positivity is demonstrated in the current study [1]. Further, the viral load of HHV-6B correlates with markers that reflect inflammatory injury. Neuroinflammation is recognized as a key component of epilepsy pathogenesis [6]. If HHV-6-related febrile seizures are involved in the etiology of temporal sclerosis and TLE, antivirals that penetrate the blood-brain barrier administered at a young age for treatment of prolonged febrile seizures could prevent the development of MTLE [6].
